# Revealing the Effects of Nanoscale Membrane Curvature on Lipid Mobility

**DOI:** 10.3390/membranes7040060

**Published:** 2017-10-18

**Authors:** Abir Maarouf Kabbani, Xinxin Woodward, Christopher V. Kelly

**Affiliations:** Department of Physics and Astronomy, Wayne State University, Detroit, MI 48201, USA; abir.kabbani@wayne.edu (A.M.K.); fq7953@wayne.edu (X.W.)

**Keywords:** fluorescence recovery after photobleaching, fluorescence correlation spectroscopy, single-particle tracking, supported lipid bilayers, membrane curvature, diffusion

## Abstract

Recent advances in nanoengineering and super-resolution microscopy have enabled new capabilities for creating and observing membrane curvature. However, the effects of curvature on single-lipid diffusion have yet to be revealed. The simulations presented here describe the capabilities of varying experimental methods for revealing the effects of nanoscale curvature on single-molecule mobility. Traditionally, lipid mobility is revealed through fluorescence recovery after photobleaching (FRAP), fluorescence correlation spectroscopy (FCS), and single particle tracking (SPT). However, these techniques vary greatly in their ability to detect the effects of nanoscale curvature on lipid behavior. Traditionally, FRAP and FCS depend on diffraction-limited illumination and detection. A simulation of FRAP shows minimal effects on lipids diffusion due to a 50 nm radius membrane bud. Throughout the stages of the budding process, FRAP detected minimal changes in lipid recovery time due to the curvature versus flat membrane. Simulated FCS demonstrated small effects due to a 50 nm radius membrane bud that was more apparent with curvature-dependent lipid mobility changes. However, SPT achieves a sub-diffraction-limited resolution of membrane budding and lipid mobility through the identification of the single-lipid positions with ≤15 nm spatial and ≤20 ms temporal resolution. By mapping the single-lipid step lengths to locations on the membrane, the effects of membrane topography and curvature could be correlated to the effective membrane viscosity. Single-fluorophore localization techniques, such SPT, can detect membrane curvature and its effects on lipid behavior. These simulations and discussion provide a guideline for optimizing the experimental procedures in revealing the effects of curvature on lipid mobility and effective local membrane viscosity.

## 1. Introduction

The shape of biological membranes is precisely controlled for diverse, essential, cellular processes, such as regulating organelle morphology, exocytosis/endocytosis, pathogen vulnerability/protection, and effective therapeutic targeting [1,2,3]. Accordingly, the dysregulation of membrane curvature is broadly implicated in cardiovascular disease, viral infections, cancer, Alzheimer's disease, Huntington disease, diabetes, and other diseases [4,5,6]. Each of these processes requires the fusion or fission of <50 nm radius vesicles with otherwise near planar membranes via precise regulation of the local curvature-generating forces [7]. Cellular membrane shape regulation incorporates a wide variety of proteins that can bend the membrane. For example, Bin/Amphiphysin/Rvs (BAR) domains have an intrinsic molecular shape [8], clathrin proteins create a scaffold [9], and intrinsically disordered proteins apply steric repulsion to induce membrane curvature [10]. The underlying non-specific, lipid-based influences remain relatively unknown in complex cellular membranes, although the importance of some key lipids has been previously demonstrated [11,12,13,14]. 

Experimental studies on the effects of curvature on membrane behavior are becoming possible with nanoengineering and super-resolution microscopy. For example, naturally occurring plasma membrane tubules [15] and engineered membrane tubules [16] have demonstrated protein and lipid sorting dependent on the membrane curvature. Nanoscale tubules have been created with model membrane via microbead pulling [17], protein crowding [18], or molecular motor pulling [19]. Prior experimental attempts to reveal diffusion differences on membrane tubules of varying radii were complicated by coupled variations in membrane composition and tension. However, slower diffusion is consistently observed on membrane tubules of smaller radii [20,21,22], as expected theoretically [23,24,25,26].

By engineering curvature on a solid substrate, modeled or living membranes may assume the substrate topography if the substrate radius of curvature and the membrane-substrate adhesion are sufficiently large. These engineered buds represent a membrane shape similar to endocytic pits preceding vesiculation and the post-fusion state of exocytosis, although the engineered structures are static, while endocytosis and exocytosis require dynamic membrane changes. Nanoscale membrane buds have been formed over substrates patterned via electron-beam lithography [16,27,28,29,30] and polystyrene nanoparticles [31,32,33,34]. These studies have revealed the effects of membrane curvature on protein sorting [16,27,34], lipid phases [28,29,30], and single-lipid dynamics [31,32,33]. 

Substrate nanoengineering has enabled the creation of the same membrane topographies simulated in this manuscript. The limited studies to date regarding the effective membrane viscosity on a nanoscale buds have reported that curvature can have no effect [32] or can slow the lipid mobility to 4–10% of the planar system [33,34,35,36]; however, these reports vary widely in the applied experimental methods. A focus of this manuscript is to demonstrate how varying observational methods can yield varying results on the effects of nanoscale curvature. Further, the collection of membrane morphologies simulated here represent a subset of the diverse membrane shapes created during endocytosis and exocytosis. 

Understanding the effects of curvature on lipid dynamics will require separately resolving the two leaflets and the variation across ≤100 nm diameter membrane buds. Within the membrane bud, there is positive and negative principal curvature and positive and negative Gaussian curvature. The molecular structure of the constituent lipids and proteins can lead to curvature-induced nanoscale molecular sorting with compositional variation laterally across the membrane or between leaflets [21,37]. No known experiments to date have been able to distinguish the effects of nanoscale positive versus negative curvature on the effective membrane viscosity. However, membrane curvature generally seems to have the net effect of increasing the local effective membrane viscosity and slowing of the lipid and protein mobility [20,21,33,34,35,36]. Accordingly, sub-diffraction-limited spatial resolution will be necessary to experimentally measure how membrane curvature influences lipid and protein dynamics.

Optical techniques are traditionally limited by diffraction to a spatial resolution of >200 nm. Nanoscopic optical methods such as direct stochastic optical reconstruction microscopy [(d)STORM], fluorescence photoactivated localization microscopy [(f)PALM], and stimulated depletion emission microscopy (STED) [38,39,40,41,42] have improved the resolution of optical microscopy to >10 nm. Many variations on these techniques have been adapted to yield fluorophore height or orientation [43,44,45,46,47,48,49,50]. In particular, polarized localization microscopy (PLM) was designed to reveal nanoscale, membrane curvature with sub-diffraction-limited resolution [33]. PLM combines single-molecule localization microscopy (SMLM) and polarized total internal reflection fluorescence microscopy (TIRFM) [11,33]. Polarized TIRFM is sensitive to membrane orientation by selectively coupling linearly polarized fluorescence excitation with lipidated indocarbocyanine dyes (i.e., DiI, DiO, DiD) that maintain their fluorescence dipole moment in the plane of the membrane [51]. Pointillist SMLM methods, such as (d)STORM, (f)PALM, and PLM provide raw data that can be interpreted for high-throughput single-particle tracking dependent on membrane curvature. Tracking individual fluorophores that stay *on* for multiple sequential frames enables the observation of single-molecule diffusion rates versus membrane topology. For example, DiI molecules diffuse on curved membranes at <10% of the speed at which they diffuse on flat membranes [33,35]. Analysis of single-molecule diffusion rates relative to membrane topology may reveal information on the local environment (i.e., lipid phases or molecular crowding) cause by membrane bending. 

In this manuscript, we demonstrate the capabilities of various fluorescence techniques to reveal lipid dynamics relative to membrane curvature. We focus on the three most common methods of measuring lipid mobility: fluorescence recovery after photobleaching (FRAP), fluorescence correlation spectroscopy (FCS), and single-particle tracking (SPT). Through Monte Carlo simulations of Brownian diffusing lipids over membrane buds of varying heights, we demonstrate the ability of each of these techniques in revealing the presence of the membrane bud, the single-molecule dynamics on the bud, and the effects of curvature on lipid mobility. Our simulations demonstrate how FRAP was not sufficiently sensitive to reveal that a bud was present under any of our simulation conditions. FCS revealed the bud’s presence, but FCS is typically limited to diffraction-limited length scales. SPT, however, measured the effects of membrane topography change with and without curvature-induced alteration to lipid mobility on each part of the membrane bud. By mapping the single-lipid steps over space, buds of varying heights and membranes of laterally varying viscosity could be distinguished. Within these simulations, we consider the effects of lipid diffusion variations with membrane curvature could have on the collected data, but we do not advocate for any particular function of curvature dependence on the diffusion rates or distinguish between the different types of lipids. Through carefully chosen methods, SPT data can reveal spatial information across the sample with <20 nm resolution. Guidelines are provided for designing SPT experiments to optimize the resolution of membrane curvature and its effects on molecular mobility.

## 2. Methods

**Figure 1:**
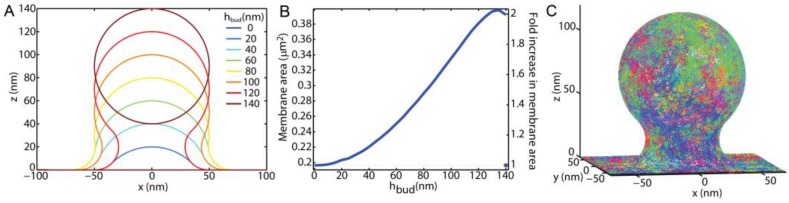
Simulations were performed by mimicking the membrane topography throughout the fission and fusion of a single 50 nm radius vesicle with a planar membrane. (**A**) By maintaining a 50 nm radius of curvature on top and a smooth, 20-nm radius of curvature connection to the surrounding membrane, the stage of membrane budding was tracked via the maximum bud height above the surrounding membrane (*h_bud_*); (**B**) Bud growth results in an increased area of total membrane within a 250 nm of the bud center. When *h_bud_* > 137 nm, the bud undergoes fission, a separate vesicle is formed, which is assumed to diffuse away and not contribute to these simulations; (**C**) Simulated trajectories are shown in assorted colors over a membrane topography with *h_bud_* = 120 nm.

The diffusion of lipids through membrane buds was simulated and analyzed to mimic the expected experimental results that would be obtained by a variety of fluorescence-based methods. All of the simulations were performed with custom MATLAB (MathWorks, Inc., Natick, MA, USA) programming, which is available in the Supplemental Material. Membrane buds were modeled with a radius of curvature equal to 50 nm and varying heights above a surrounding planar membrane (*h_bud_*). The bud membrane was smoothly connected to the surrounding planar membrane with a radius of curvature equal to 20 nm along the principal plane radial from the bud center (Figure 1), as done previously [33,34]. *h_bud_* = 0 represents the case of a planar membrane with no bud protrusion. When *h_bud_* = 140 nm, the bud had detached from planar membrane such that there was no diffusion between the vesicle and the planar membrane, and the vesicle was assumed to not contribute to the observed lipid diffusions as if the newly formed endosome had quickly left the proximity of the plasma membrane. Simulations of diffusion on a vesicle disconnected from a surrounding SLB have been recently published [35]. This is a minimalistic model of membrane shape during endocytosis or exocytosis that qualitatively matches electron microscopy images of cell plasma membranes [3,16] and allows for the single parameter *h_bud_* to report the stage of progression as the membrane transitions between a plane and the highly bent moment of fission or fussion. 

The trajectories of the individual lipids were simulated upon the budding topography via a Monte-Carlo method for a discrete set of randomly distributed points. The discrete points were created at a density of 4 points/nm^2^ across the bud top, the bud-to-planar membrane neck, and the surrounding planar membrane. At each time step, the lipid moved to one of the 110 ± 10 random points within 3 nm. This resulted in an average single step distance of 2 nm. To mimic the diffusion coefficient (*D*) of 1 μm^2^/s over many steps, each time step would correspond to 1.1 μs. Each trajectory of each lipid started 1 μm away from the bud center, then diffused randomly upon the simulated membrane until it was >1 μm away from the bud center. More than 10^5^ different trajectories were simulated for each condition, and 1300 ± 100 of those trajectories made it onto the membrane bud for each *h_bud_*. Example trajectories over a bud of *h_bud_* = 120 nm are shown in Figure 1C. 

To mimic curvature-induced slowing of lipid diffusion, the effective time per simulation step was changed to be 11 μs or 28 μs for each 2 nm step whenever the simulated lipid was on the bud to mimic *D_Bud_* = 0.1 or 0.04 μm^2^/s, respectively. Simulations were performed with the varying values of *D_Bud_* to reveal how the various observation methods would report curvature-induced lipid slowing.

The analyses performed in this manuscript were limited to the *z*-projection of the fluorescence signal into the imaging *xy*-plane. The fluorescence emission was assumed to have no *z*-dependence or polarization dependence. These two assumptions apply well to topographies that vary in thickness by less than the diffraction-limit and for fluorophores that tumble randomly through all of the orientations, respectively. 

### 2.1. Mimicking Fluorescence Recovery after Photobleaching

Fluorescence recovery after photobleaching (FRAP) measures the recovery of a fluorescence signal after the fluorophores within a region of the sample were bleached. Here, two bleaching conditions were used and compared. The first bleaching method, “complete bleaching”, was identical to the processes of coming to equilibrium, as described above, in which all the fluorophores within 1 μm from the bud center were bleached. The second method, “Gaussian bleaching”, took an equilibrated distribution of fluorophores and imposed a bleaching probability upon the *i*th fluorophore equal to $P_{GB}\left(i\right)=\;\exp\left(-2r_{i}^{2}/w^{2}\right)$ with a *w =* 250 nm and r_i_ equal to the lateral distance of the fluorophore from the bud center. In both cases, simulated trajectories were analyzed with a Gaussian illumination (*I*) centered on the membrane bud according to

(1) $$I\left(t\right)=\Sigma_{i}\;\exp\left(-2r_{i}^{2}/w^{2}\right)$$

with an illumination width (*w*) set equal to 250 nm, as would be expected for a diffraction-limited illumination. Upon Gaussian bleaching, *I*(t) was reduced to 50 ± 5% of the equilibrated intensity.

New single-lipid trajectories were started at the perimeter of the 2 μm diameter system every 0.08 μs for an equilibrium density of 0.00146 lipids/nm^2^ or approximately 10 mol % fluorescent lipids. Since an area larger than the illumination spot was initially bleached with the complete bleaching method, only the *I*(*t*) after the 25% of the recovery had occurred was analyzed. The increasing *I*(*t*) was fit to

(2) $$I_{FRAP\;Fit}\left(t\right)=\;A\left(1-e^{-t/\tau_{FRAP}}\right)$$

The fitting variable *A* represents the steady-state magnitude of *I* and is proportional to the steady-state fluorophore density, the membrane area, the illumination intensity, and the fluorescence emission collection efficiency. The fitting variable *τ_FRAP_* represents the characteristic FRAP recovery time. This fitting model is an approximation to the expected recovery for planar systems with uniform [52] or Gaussian illumination [53]; this approximation was used since the exact recovery shape over the non-planar membrane topography would with *h_bud_*, which would not be typically known a priori.

### 2.2. Mimicking Fluorescence Correlation Spectroscopy

Fluorescence correlation spectroscopy (FCS) examines the fluctuations in the steady state *I* versus *t* signal. This is performed by calculating the autocorrelation (*G*) as a function of lag time (*τ*) and finding the characteristic fluctuation time (*τ_FCS_*). In these simulations, *I*(*t*) for FCS was calculated from the single-molecule trajectories through a Gaussian illumination profile according to Equation (1), as would be expected for typical confocal FCS. *G* was calculated from *I*(*t*) according to

(3) $$G\left(\tau \right)=\;<\delta I\left(t\right)\delta I\left(t-\tau \right)>/<I\left(t\right){>}^{2}$$

The angle brackets (< >) represent the average over *t* and *δI*(*t*) = *I*(*t*) − <*I*(*t*)>. The correlation time (*τ_FCS_*) in *I*(*t*) was found by fitting *G*(*τ*) according to

(4) $$G_{Fit}\left(\tau \right)=G_{0}{\left(1-{\left(\tau /\tau_{FCS}\right)}^{2}\right)}^{-1}$$

as is expected for two-dimensional (2D) Brownian diffusion. The fitting variable, *G*_0_, is inversely proportional to the number of diffusers simultaneously observed and other experimental conditions not relevant in these simulations. With a membrane bud present, the autocorrelation is not expected to fit perfectly to Equation (4); however, the inherent averaging incorporated into an autocorrelation analysis makes finding minor populations difficult, and complex fitting functions are typically unwarranted [54]. For this analysis, Equation (1) was assumed to also be the spatial detection sensitivity, which is the standard approximation of the Airy point spread function in a diffraction-limited system [55] and especially accurate when a confocal collection pinhole is used with a diameter of one Airy unit.

### 2.3. Mimicking Single Particle Tracking

Single-particle tracking (SPT) includes identifying the center of each single-fluorophore image via computational analysis of a movie of sparse, dynamic fluorophores. From the motion of the single-molecules between sequential frames, single-molecules trajectories were observed. The single-steps lengths (*s*) observed in a region of the sample may be fit to a 2D Maxwell-Boltzmann or Rayleigh Distribution to determine the local *D*, where the probability distribution (*P*) of step lengths over a time step of Δ*t* for a single Brownian diffuser in a uniform membrane is expected to be

(5) $$P\left(s\right)=\frac{s}{2D\Delta t}e^{-\frac{s^{2}}{4D\Delta t}}$$

The observed single-molecule steps were grouped and fitted according to their location in the sample (*x*, *y*) or distance from the bud center (*r*) such that *D* could be measured at different locations in the sample. The ability to gain spatial resolution for variations in *D* across a sample is the key differences between a single-step length analysis used here versus the more traditional mean squared displacement (*MSD*) analysis, as discussed below. Optimization of MSD analysis has been recently described [56].

However, the single-step analysis in experimental systems is affected by the 2D localization uncertainty ($\sigma_{r}^{2}=\sigma_{x}^{2}+\sigma_{y}^{2}$) and the camera exposure duration (*t_exp_*) to yield a systematic difference between the *D* found from fitting Equation (5) to experimental data (*D_Fit_*) and the *D* that would be found from an idealized system (*D_Real_*), according to

(6) $$D_{Real}=\left(D_{Fit}-\frac{\sigma_{r}^{2}}{2\Delta t}\right)/\left(1-\frac{t_{exp}}{3\Delta t}\right).$$

Frequently, Δ*t* is equal to the time between adjacent frames (*t_frame_*) used in this analysis, however, any Δ*t* that is a multiple of *t_frame_* are permitted. *t_frame_* is equal to the sum of t_exp_, and the frame read time such that the inverse of 1/*t_frame_* equal the imaging frequency.

When the membrane is not parallel to the coverslip, then the *z*-component of the lipid diffusion within the membrane, results in a slowing of the lipid through the *xy*-plane. It was not possible to extract the in-plane diffusion rate from the observed diffusion through the *xy*-plane (*D_xy_*), when both the membrane topography and the influence of curvature on membrane viscosity are unknown. In the below analysis, *D_Real_* was calculated under the approximation that the membrane was parallel to the coverslip, and this value was reported as *D_xy_* to be explicit in representing that the membrane topography was contributing to the measurements. 

### 2.4. Assumptions Used in the Simulations

Super-resolution methods may incorporate a light polarization and *z*-dependence in the localization probability versus membrane height or orientation. The localization probability can vary as a fluorophore diffuses away from the objective focal plane in the *z*-direction. However, this probability change occurs only when variations in *z* are greater than the diffraction limit are allowable. Since the nanoscale buds simulated here vary in *z* by <140 nm, no variation in the probability of fluorophore localization is expected. Polarization was important in FCS of vesicles containing DiD [35]; however, no differences in s- versus p-polarized illumination was observed in SPT of DiI [33]. The simulations presented here mimic randomly tumbling fluorophores with no polarization dependence.

Simulations performed here do not incorporate any differences in the lipid diffusion due to the membrane-substrate adhesion. The diffusion of transmembrane proteins and >100 nm diameter lipid domains can be greatly slowed in SLBs versus freestanding membranes; however, the diffusion of individual lipids are significantly less affected by the substrate [57]. We would expect to see significant substrate effects for fluorescent lipids with larger head-group labeling [58] or for systems that may form >100 nm phase-separated domains.

## 3. Results

### 3.1. Membrane Bending and FRAP

**Figure 2:**
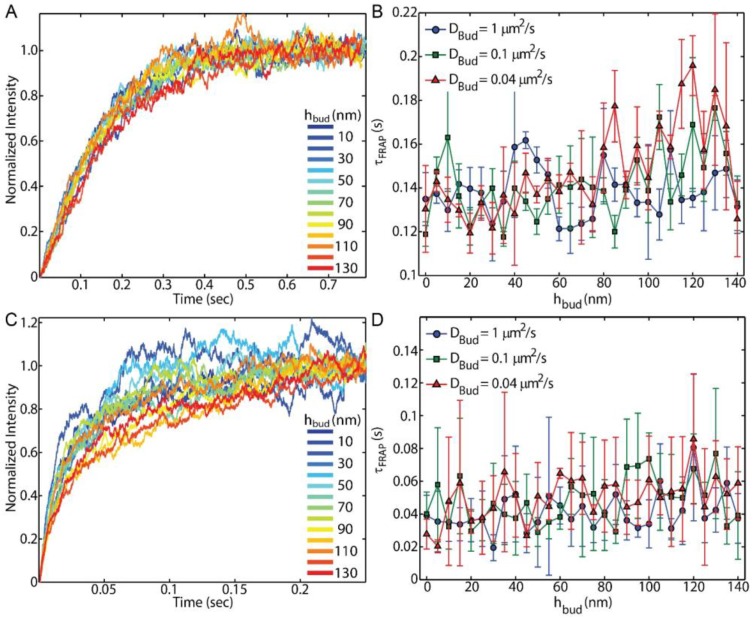
Increasing the bud height does not result in significant changes to the fluorescence recovery after photobleaching (FRAP) recovery time, including when the lipid diffusion was slowed to *D_Bud_* = 0.1 or 0.04 μm^2^/s on the curved membrane for both (**A**,**B**) complete bleaching and (**C**,**D**) Gaussian Bleaching; (**A**,**C**) Simulated *I*(*t*) traces while *D_Plane_* = 1 μm^2^/s and *D_Bud_* = 0.1 μm^2^/s shows the recovery of *I*(*t*) after bleaching. There was no apparent trend in the recovery rate changing with bud height; (**B**,**D**) The recovery rate was quantified by fitting Equation (2) to find *τ_FRAP_* from *I*(*t*) of each condition. Error bars represent the standard error of the mean between four repeated simulations.

Experimental FRAP conditions, such as the laser-based illumination with complete or Gaussian bleaching and small observation areas, were mimicked in the simulations presented here. The presence of the membrane bud and the extra membrane area associated with it were not sufficient to consistently influence the FRAP recovery time. Upon incorporating a curvature-induced slowing of the lipid diffusion on the bud, where *D_Bud_* was reduced to 0.1 or 0.04 μm^2^/s, while maintaining *D_Plane_* = 1 μm^2^/s, the variation between repeated simulations proved to provide insignificant differences in τ*_FRAP_* versus *h_bud_* for both complete bleaching (Figure 2A,B) and Gaussian bleaching (Figure 2C,D). Further, these results were created from a perfectly centered Gaussian illumination on the bud center (Equation (1)). Upon slight deviations of the illumination from the bud center, the effects of the bud on τ*_FRAP_* were reduced further. Within the range of *D_Bud_* explored here, the identification of the bud and the curvature-induced changes in lipid diffusion were undetectable by FRAP.

### 3.2. Membrane Bending and FCS

**Figure 3:**
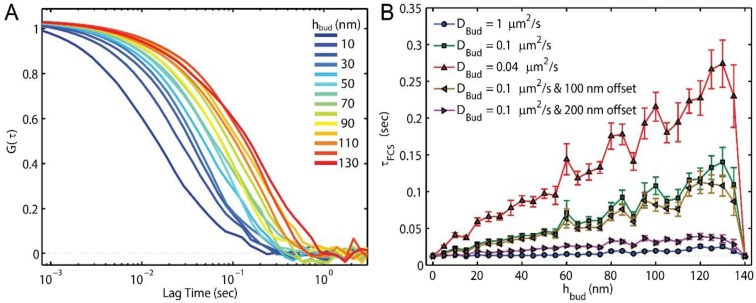
A membrane bud may be detected by fluorescence correlation spectroscopy (FCS) if *h_bud_* > 80 nm or the membrane curvature slowed the local lipid diffusion. While a lipid on the planar membrane experiences *D_Plane_* = 1 μm^2^/s, varying the diffusion rate of the lipid on the curved membrane and the location of the bud within the excitation spot affects the observed FCS results. (**A**) A shifting of *G*(*τ*) to longer lag times was apparent when *D_Bud_* = 0.1 μm^2^/s and the bud was centered on the 250 nm wide Gaussian illumination; (**B**) If *D_Bud_* = 1 μm^2^/s and *h_bud_* ≤ 50 nm, a minimal effect of the bud was observed on the measured *τ_FCS_*; the extra membrane area and varying membrane orientation are not sufficient to affect the FCS results within the uncertainty of finding *τ_FCS_*. However, if *D_Bud_* = 0.1 μm^2^/s, the presence of the bud becomes clear for *h_bud_* ≥ 15 nm. The bud can be offset by 200 nm from the center of the illumination and still yield a clear change in the *τ_FCS_*. If the observation spot was off-centered from the bud by 100 nm or 200 nm, the effects of the bud are decreased by an average of 15% or 66%, respectively.

The presence of a single nanoscale membrane bud could be detected with FCS with or without curvature-induced lipid slowing (Figure 3). With diffraction-limited illumination (Equation (1)) and imprecise centering of the observation spot over the membrane bud, simulated FCS detected the effects of the bud on the lipid dwell time. For example, with *D_Plane_* and *D_Bud_* = 1 μm^2^/s, the addition of the extra membrane area for a bud of *h_bud_* = 100 results in a 60% slowing of the intensity fluctuations; *τ_FCS_* = 12.3 ± 0.6 ms and 20 ± 1 ms when *h_bud_* = 0 and 100 nm, respectively. This change is consistent with the 60% extra membrane area created by the bud when *h_bud_* = 100 nm versus 0 nm (Figure 1B). 

When the membrane bud incorporated slower lipid diffusion, the effects of the membrane bud on the FCS result were more pronounced. For example, a 1700% slowing *τ_FCS_* was observed between *h_bud_* = 0 and 100 nm when *D_Bud_* = 0.1 μm^2^/s and *D_Plane_* = 1 μm^2^/s, with *τ_FCS_* = 12.3 ± 0.6 ms and 210 ± 20 ms, respectively; the autocorrelations for varying *h_bud_* are shown for this condition in Figure 3A.

The effects of the membrane bud on the FCS results were apparent but reduced if the fluorescence illumination was not well centered over the membrane bud. For example, the effects on *τ_FCS_* was decreased by an average 15% when the center of the membrane bud was 100 nm offset away from the center of the diffraction-limited FCS illumination spot, as calculated by the average ratio of the centered versus offset *τ_FCS_* values for *h_bud_* from 5 nm to 135 nm (Figure 3B).

### 3.3. Membrane Bending and SPT

**Figure 4:**
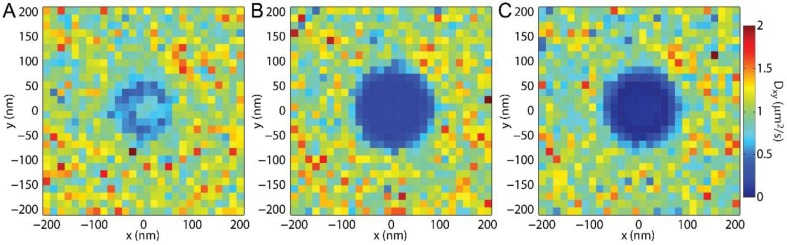
*D_xy_* mapped over the sample through single particle tracking (SPT). The single steps from all trajectories are binned according to the average *x* position and the average *y* position of the two localizations. This two-dimensional (2D) binning of the *x* and *y* positions allows for the analysis of all the step lengths in each region on the sample, as represented by a single pixel in these images. Here, *h_bud_* = 100 nm, *σ_r_* = 15 nm, *D_Plane_* = 1 μm^2^/s, and *t_frame_* = 2 ms while (**A**) *D_Bud_* = 1 μm^2^/s; (**B**) *D_Bud_* = 0.1 μm^2^/s; and (**C**) *D_Bud_* = 0.04 μm^2^/s. The bud induced slowing in (**A**) was due to the membrane topography causing the lipid to move slower through the *xy-*plane with constant local diffusion in the membrane of varying orientation; this effect also contributes to (**B**,**C**). The distinction between the bud and the planar membrane is enhanced when there is a greater difference between *D_Plane_* and *D_Bud_*.

**Figure 5:**
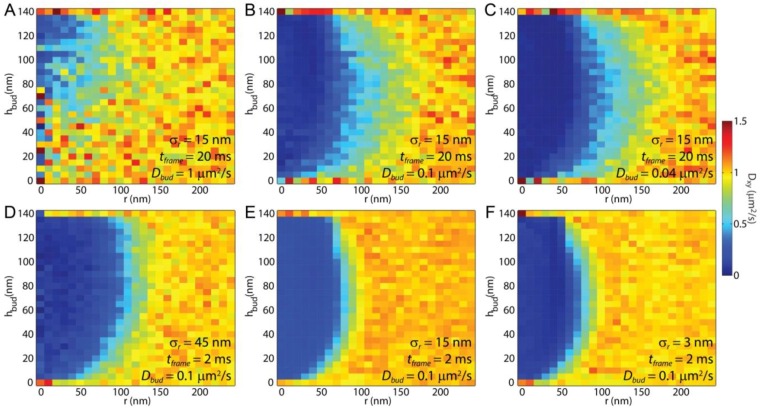
Azimuthal averaging of the spatial mapping of *D_xy_* around a bud (i.e., Figure 4) improved the data statistics and enabled easier comparison of *D_xy_* versus distance from the bud center (*r*) and *h_bud_*. Simulated SPT results for *D_xy_* are presented with varying *σ_r_*, *t_frame,_* and *D_Bud_*. (**A**) When *D_Plane_* = *D_Bud_* = 1 μm^2^/s and, the single molecules maintain a uniform average local speed through the membrane, and the observed variations in *D_xy_* were due to the tilt of the membrane; (**B**,**C**) As *D_Bud_* decreased to 0.1 or 0.04 μm^2^/s, the bud became increasingly apparent at all *h_bud_*; The (**D**–**F**) significant improvements in the resolution of *D_xy_* were obtained by decreasing *t_frame_*, even at the expense of increasing *σ_r_*; (**F**) Differences in Dxy may be observed across the bud, including slowing at the vertical edge of the bud.

Mapping the locations of single molecule steps enables measuring *D_xy_* at specific locations within the sample (Figure 4). When a membrane with consistent viscosity and consistent lipid mobility was simulated, the variations in the measured *D_xy_* across the sample were due to the membrane topology change, and the measured shorter lipid step lengths on tilted membranes as the lipid trajectories are projected on the imaging *xy*-plane (Figure 4A). The effects of the membrane bud are enhanced when the curved membrane reduces the lipid mobility (Figure 4B,C). When the membrane bending occurs in a rotationally symmetrical way, as would be expected for an endocytic pit and the engineered curvature shown here, a radial averaging provides greater clarity in the effects of membrane budding on the SPT results (Figure 5). 

In an SPT experiment, a balance between acquisition frame rate, localization precision, laser power, and *on* fluorophore density is required to achieve meaningful data. The simulations performed here demonstrate that a faster frame rate at the cost of worse localization precision yields.

A finer resolution of the lipid mobility for most experimental conditions. The most common experimental SMLM and SPT parameters result in *σ_r_* = 15 nm and *t_frame_* = 20 ms. The effects of the membrane bud for the membrane topography simulated here, assuming *D_Plane_* = *D_Bud_,* resulted in a negligible change in *D_xy_* versus *r* until *h_bud_* ≥ 60 nm (Figure 5A). The greatest loss of signal with these conditions came from the long distance the fluorophore moved between adjacent frames, and individual lipids sampling both the curved and planar portions of the membrane within a single step. For *t_frame_* = 20 ms and *D* = 1 μm^2^/s, the expected step length of a single fluorophore between adjacent frames (${\bar{s}}_{1}$) would be (4*Dt_frame_*)^1/2^ = 280 nm. This is much farther than noise added by the single-fluorophore localization imprecision.

When the membrane curvature caused the local lipid diffusion coefficient to slow, the membrane bud was readily apparent via SPT with *σ_r_* = 15 nm and *t_frame_* = 20 ms for any *h_bud_* (Figure 5A–C). SPT revealed *D_xy_* for the top of the bud separate from the surrounding planar SLB; however, the diffusion around the bud neck was challenging to interpret when considering the combined effects of membrane tilt and data blur. 

Significant benefits can be obtained from SPT results by employing faster imaging frame rates. Keeping all other parameters constant, the effects of decreasing *t_frame_* from 20 ms to 2 ms resulted in a 3x improvement in blur induced by diffusion between frames (Figure 5B,E). Additionally, the shorter *t_frame_* provided an improvement in data statistics for a given total acquisition time, this is mainly due to the increased number of independent steps observed per time. Even if decreasing *t_frame_* comes at the cost of increasing *σ_r_* to 45 nm, there was still a clear improvement in the obtained results (Figure 5B,D). This relative importance of *t_frame_* and *σ_r_* is further demonstrated by showing how marginal improvements in the resolution of *D_xy_* were obtained if *t_frame_* = 2 ms and *σ_r_* was decreased from 15 nm to 3 nm, which is feasible when fluorescent beads or metal nanoparticles are used for tracking rather than single fluorophores (Figure 5E,F).

## 4. Discussion

Resolving the nanoscale biophysical effects of membrane curvature remains experimentally challenging. Optical techniques such as PLM enable the detection of curvature with higher sensitivity and resolution than conventional optical techniques, while providing a biologically friendly imaging environment. However, decoupling the effects of varying membrane area, orientation, and curvature can be challenging when the primary data collected is the *z*-projection of sample topography changing on sub-diffraction-limited length scales. The focus of this manuscript is to provide a framework for protocols and capabilities of experimental measurements of lipid diffusion as influenced by nanoscale membrane curvature. The simulation results presented here guide the experimental procedures that will improve the understanding of membrane curvature.

### 4.1. Capabilities of FRAP, FCS, and SPT

The method of draping a supported lipid bilayer over nanoparticles has been used in prior experimental studies to reveal the influence of curvature on lipid dynamics and protein sorting. FRAP [30,31] and SPT [32,33] have been used to confirm the continuity of the membrane over the nanoparticles. FRAP was used to confirm membrane integrity by matching the final fluorescence emission intensity after bleaching and recovery to the before-bleaching intensity. SPT was used to confirm membrane integrity through the observation of single-particle trajectories connecting the bud to the surrounding SLB. However, FRAP was unable to reveal any difference in the recovery rate of the fluorescence due to the presence of curvature in experiments [31,32], as expected by the simulations performed here (Figure 2). The setup in these simulations matches most prior experimental approaches where a single membrane bud is being observed at a time. Should the bud density become high enough for multiple buds were present within the FRAP observation region, FRAP may prove to provide bud-dependent results.

FCS can detect the effects of the bud and curvature-induced slowing on lipid diffusion in these simulations with diffraction-limited illumination (Equation (1)) and imprecise centering of the observation spot over the membrane bud (Figure 3). However, the ability for FCS to detect membrane buds without curvature-induced lipid slowing was limited to mature buds (*h_bud_* > 55 nm) for a 20% change in *τ_FCS_*. With curvature induced slowing (*D_Bud_ ≤ D_Plane_*/10), significant changes to *τ_FCS_* are apparent as soon as the bud is formed. Additionally, it would be feasible to provide sub-diffraction-limited resolution of the bud’s location in the sample without a complementary signal (e.g., nanoparticle, atomic force microscopy, or clathrin co-localization) by finding where τ*_FCS_* was most slowed when scanning the illumination beam over the sample. Without a complementary signal co-localized to the slowed *τ_FCS_*, however, it would be difficult to confirm that *τ_FCS_* slowing was caused to a membrane budding rather than another membrane defect, such as membrane-substrate interaction or sorting to domains of lipid phase separation.

FCS is a valuable technique for identifying the presence of a membrane bud and/or the magnitude of slowing induced by the curvature; however, the ability of FCS to reveal the lipid mobility on different parts of the bud are prohibited by the relatively large size of the diffraction-limited illumination (*w* = 250 nm) as compared to the bud radius (50 nm). Potentially, sub-diffraction-limited STED-FCS could yield a greater resolution of the lipid mobility on distinct parts of the bud as well as increase sensitivity to the membrane topography itself [59]; however, STED-FCS is technically challenging, expensive, and rare. Sub-bud resolution may be achievable with FCS by exploiting rotationally-limited diffusion of fluorophores with polarized excitation or emission, as has been done on lipid vesicles of varying size [35].

SPT is unique among these techniques in that it provides sub-diffraction-limited spatial resolution with the capability to reveal the locations on the bud that most affect the local lipid diffusion. By binning the single-lipid step size versus distance from the center of the membrane bud and fitting the resulting histogram of step lengths to the Rayleigh distribution, the effects of lipid topography can be revealed directly even without curvature-affected lipid mobility (Figure 4A and Figure 5A). The effect of the membrane bending was observed via *D_xy_* in these simulations without any curvature-induced changes to *D*. Similarly, observation of *D_xy_* across a sample could reveal previously unknown membrane topography if there were curvature induced change to lipid mobility were known. This is particularly shown in Figure 4C, in which the vertical edge of the membrane bud, the horizontal top of the bud, and the surrounding SLB are each individual identifiable.

### 4.2. Comparative Ease of Performing FRAP, FCS, and SPT

The decision of which technique to use requires considering both the type of information needed to be obtained and the associated technical challenges. FRAP, FCS, and SPT each provide benefits regarding the specific membrane processes that they reveal and the ease by which they are experimentally performed. FRAP is the easiest to these techniques in both the execution and analysis of the experiment. FRAP can be carried out on large observation regions with a conventional epifluorescence microscope through the opening and closing of a field diaphragm in the conjugate image plane. Alternatively, greater spatial resolution can be gained with diffraction-limited bleaching and illumination, as simulated here. Although these methods of performing FRAP can provide a coarse analysis of membrane integrity and lipid mobility, it can be difficult to achieve a high intensity of the fluorescence emission and small enough observation areas to provide precise measurements. Even if performed with a relatively weak and slow bleaching procedure, FRAP can reveal the fraction of the diffusers that are immobile and the average diffusion coefficient of the mobile diffusers in a large observation area. This is especially valuable for demonstrating the continuity of a model membrane.

The correlation of intensity versus time for FCS only reports the diffusers that move through the diffraction-limited observation spot over the ~30 s of data collection; FCS does not incorporate any information from immobile particles on a membrane. However, FCS is more sensitive to sub-populations of diffusers than FRAP and provides greater accuracy in the measured diffusion coefficients of the mobile diffusers. Commercial FCS setups require laser illumination, expensive detectors, hardware correlators, and software, but can provide analysis of the results in real-time. Homebuilt FCS setups may use high-frame rate EMCCD or sCMOS cameras and custom software for correlation calculation and fitting. Since FCS can reveal late-stage bud formation and the effects of bending on lipid mobility, it is feasible that future incorporations of FCS will be used to report the biophysical ramifications of membrane bending.

As shown above, SPT provides the best spatial resolution of membrane bending and the effects of bending on lipid mobility. However, SPT requires significantly more effort in data collection and analysis. SPT requires a high photon flux of the emission (>5000 photons/s) for precise single-fluorophore fitting (*σ_r_* < 50 nm) with fast frame rates (≥50 Hz), which often requires oxygen-scavenging buffers to reduce fluorophore oxidization to provide more photons per fluorophore ‘on’ state and greater conversion from the fluorophore ‘off’ to ‘on’ states. The raw SPT data typically comprises ≥5000 individual camera images, from which the single-molecule locations are calculated. The locations are linked for trajectory analysis, MSD analysis, and/or single-step length analysis. A complicating factor of SPT is that the data quality and signal-to-noise ratio can vary between experiments, such that user confirmation is needed for the analysis of each experiment. Despite these experimental challenges, the resolution benefits of SPT commonly justify its implementation. 

### 4.3. SPT without Long Trajectories

Typically, the single-particle trajectories are analyzed by calculating the MSD versus Δ*t* such that 2D Brownian diffusion results in a linear relationship of

(7) $$MSD\left(\Delta t\right)=\;4D\Delta t+\;2\sigma_{r}{}^{2}-8DRt_{frame}$$

The localization uncertainty and camera blur contribute to these last two terms of Equation (7), respectively. Camera blur is a spreading of the acquired image due to the motion of the subject during the finite single-frame acquisition time. Camera blur depends on the motion blur coefficient (*R*), such that *R* = *t_exp_*/(6*t_frame_*) ≤ 1/6 for continuous exposures within each frame [60]. *MSD* analysis has the potential to provide a precise *D* for a single diffuser and prior analysis has optimized the experimental parameters for *MSD* analysis [56]. However, *MSD* versus Δ*t* analysis spatially averages each trajectory by providing a single *D* for all the space explored during the single trajectory. For trajectories that include dozens of sequential localizations, this analysis may provide an average over many square microns of the sample, well beyond the extent of a membrane bud. Conventional *MSD* analysis is unable to provide the curvature-dependent diffusion of lipids when only a small fraction of each trajectory is on the membrane bud. 

### 4.4. Effects of Frame Rate on SPT

Individual fluorophore localizations for SMLM or SPT are frequently made to ≤20 nm certainty with precisely optimized imaging buffers, illumination intensities, and detection optics. However, the spatial resolution of the mobility measurement is often determined by the distance between sequential localizations rather than the precision of single localizations. SMLM methods yield *σ_r_* that is approximately proportional to the inverse of the square root of the number of photons collected per single fluorophore image (*N*), while *N* is proportional to *t_exp_* [61]. Similarly, ${\bar{s}}_{1}\;$ is proportional to $\sqrt{t_{frame}}$. Assuming minimal image readout time, minimal background noise, and *t_exp_* ≈ *t_frame_*, ${\bar{s}}_{1}\;$and *σ_r_* are approximately inversely related to each other, and an imaging frame rate could be found that results in ${\bar{s}}_{1}\;$≈ *σ_r_*, for which the best resolution of *D* across a sample could be achieved. This is demonstrated by comparing panels B and D in Figure 5 for which a substantial improvement in the resolution of *D* results from $t_{frame}\;$being decreased by 90% (from 20 ms to 2 ms), ${\bar{s}}_{1}$ decreasing by 70% (from 280 nm to 90nm), and *σ_r_* increasing by 200% (from 15 nm to 45 nm). Presumably, decreasing *t_frame_* further would have resulted in even greater resolution benefit until ${\bar{s}}_{1}$ equaled *σ_r_*; however, experimental realities, such as image background noise, becomes significant at shorter *t_frame_* when *N* < 100 such that *σ_r_* increases significantly faster than $1/\sqrt{N}\;$ and there are no longer benefits of decreasing *t_frame_*. 

Increasing the photon flux can improve both *σ_r_* and ${\bar{s}}_{1}$. For example, single biomolecules labeled with metal nanoparticles have yielded more fluorescence emission to demonstrate membrane hop diffusion with *σ_r_* = 17 nm and *t_frame_* = 0.03 ms [62]. Metal nanoparticles detected in a non-fluorescence, interferometric microscope have yielded *σ_r_* = 1.7 nm and *t_frame_* = 1 ms [63]. However, these experiments with ≤1 ms frame rates depend on >20 nm diameter gold nanoparticle labels and are associated experimental uncertainties that are not present with single-fluorophore labels. The uncertainties associated specifically with gold nanoparticle labels include the reduced specificity of the number of lipids per nanoparticle, the effects of drag on the nanoparticle, the non-specific binding between the nanoparticle and the other membrane components, and the local heating that may be caused by the gold absorption of the illumination.

## 5. Conclusions

There are numerous challenges for observing the effects of membrane bending at physiological length scales. Diverse super-resolution optical techniques are providing resolution of nanoscale membrane topography; however, the dynamical effects of curvature remain largely unknown. The ability for super-resolution optical techniques such as PLM to reveal nanoscale membrane bending is expanding the experimental capabilities for membrane curvature detection. The capacity to engineer membrane bending through the creation of supported lipid bilayers draped over nanoengineered substrates allow for the experimental creation of membrane topographies that are analogous to endocytosis and exocytosis. With SPT, the spatial resolution of lipid mobility and membrane bending can be observed with a higher precision than detectable with FCS or FRAP. The fitting of the histogram of single-step sizes distribution enables the calculation of the lipid diffusion coefficients that are corrected for the localization uncertainty and the camera blur. Future experimental implementations of PLM and SPT will reveal the effects of membrane bending on the membrane viscosity and lipid mobility. Through asymmetric labeling of model membranes, the specific contribution of each bilayer leaflet will be determined in the nanoscale budding membrane. The sum of these results will contribute to the greater understanding of membrane biophysics and the mechanisms of cellular regulation of membrane topography.

## Supplementary Materials

The custom MATLAB scripts used in this work are available online at www.mdpi.com/2077-0375/7/4/60/s1.
